# Supplementary material for: Dormant Metastases Exhibit a Unique Phenotype Primarily Promoted by the Ch25h Gene and Are Maintained in Dormancy by T Lymphocytes
Source: MedComm (2020). 2025 Oct 26;6(11):e70437. doi: 10.1002/mco2.70437 (PMC12554783; doi:10.1002/mco2.70437)
Supplement: Supplementary file 1 — Figure S1: Complete gene expression at the transcriptional level of all metastatic cell lines, obtained by mRNA sequencing. The results show the great similarity between the two cell lines that form each metastatic group. Furthermore, the dormant and nude metastatic groups are found to be part of the same cluster as B11, while the overt metastases are identified as belonging to a distinct cluster. (A) Pearson correlation between simples. (B) Cluster analysis of differentially expressed genes. (C) Sample clustering dendrogram. (D) Venn diagrams of differentially expressed genes. Dormant metastasis (MB11_Dor 1 and MB11_Dor2), nude metastasis (MB11_Nu1 and MB11_Nu2), and overt metastasis (MPA72_2A and MPA73_2A). Figure S2: The gene‐pathway enrichment in dormant metastases. Gene ontology (GO) function enrichment of the gene found to be differentially expressed in dormant metastases: 92 genes of mRNA sequencing (A); cholesterol synthesis pathway gene(B); chemokine gene (C); and surface marker genes (D) (Metascape). Figure S3: Flow cytometry gating strategies: a CD3+ x (CD4− CD8−) x TCRγδ+ gate was applied to select γδ T lymphocytes (A); CD3+ x CD4+ y CD3+ x CD8+ to classify CD4 and CD8 T lymphocytes (B); CD45+ x CD3+ y CD45+ x CD19+ to separate the T and B lymphocytes (C); CD45+ x CD3‐ x CD49b+ y CD45+ x CD3+ x CD49b+ to differentiate between NK and NKT cells (D); CD45+ x CD11b+ x LY6G+ for selecting neutrophils (E), gate showing as a representation of eosinophils (CD11b+ x SIGLEC‐F+), monocytes (CD11b+ x LY6C+), macrophages (CD11b+ x MHC‐II+), interstitial macrophages (CD11b+ x F4‐80+), alveolar macrophages (CD11c+ x SIGLEC‐F+) and dendritic cells (CD11c+ x MHC‐II+), in which the same gating strategy has been used but with its specific markers; and CD45+ x CD11b+ o CD11c+ x (LY6G− LY6C−) to identify rare myeloid cells (F). Figure S4: Relationship between CH25H expression and the expression of overexpressed genes in the dormant metastasis group in human breast cancer. TC [file MCO2-6-e70437-s001.docx]

Supplementary information for:

**Dormant metastases exhibit a unique phenotype primarily promoted by the Ch25h gene and are maintained in dormancy by T lymphocytes**

**Running Title:** The immune-controlled dormant metastasis phenotype

Virginia Chamorro^1,2^, Ignacio Algarra^3^, Verónica Sanz^1,2^, María Pulido^1,2^, Irene Romero^4^, Estefanía Chico^1,2^, Marina Millán^1,2^, María Escaño-Maestre^1,2^, Pablo Botella^1,2^, Isabel Linares^1,2^ and Ángel M. García-Lora^1,2,5
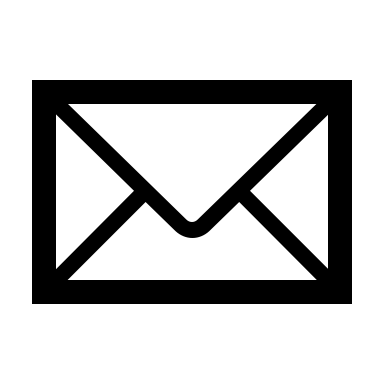
^

^1^Servicio de Análisis Clínicos e Inmunología, UGC Laboratorio Clínico, Hospital Universitario Virgen de las Nieves, Granada, Spain.

^2^Instituto de Investigación Biosanitaria ibs.GRANADA, Granada, Spain

^3^Departamento de Ciencias de la Salud, Universidad de Jaén, Jaén, Spain

^4^Servicio de Análisis Clínicos, Hospital de Antequera, Málaga, Spain

^5^Plataforma Biobanco ibs.Granada, Nodo Granada, Granada, Spain

**Supplementary materials**

**Supplementary Figures and Figure legends**


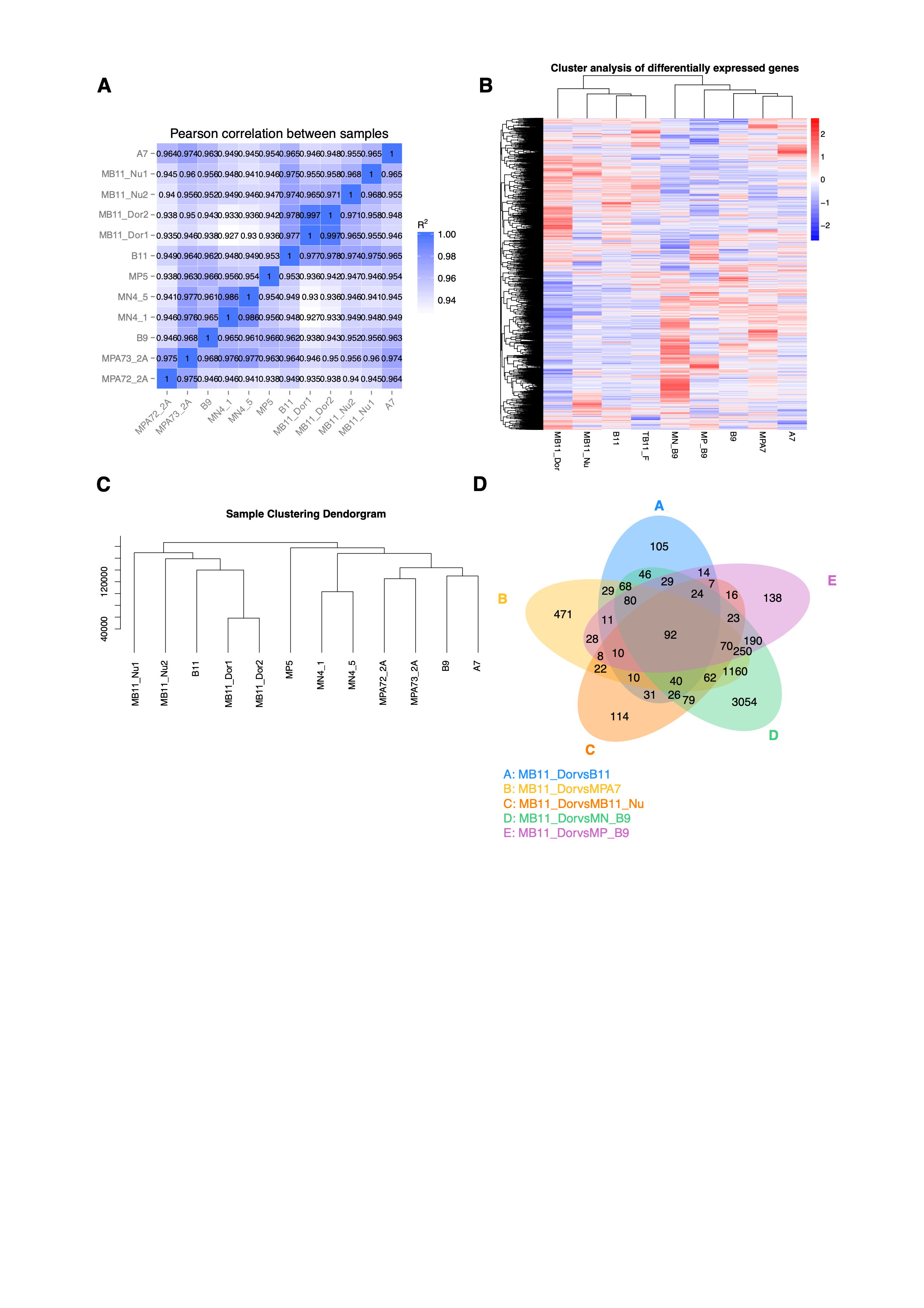


**Figure S1. Complete gene expression at the transcriptional level of all metastatic cell lines, obtained by mRNA sequencing**. The results show the great similarity between the two cell lines that form each metastatic group. Furthermore, the dormant and nude metastatic groups are found to be part of the same cluster as B11, while the overt metastases are identified as belonging to a distinct cluster. (A) Pearson correlation between simples. (B) Cluster analysis of differentially expressed genes. (C) Sample clustering Dendrogram. (D) Venn diagrams of differentially expressed genes. Dormant Metastasis (MB11_Dor 1 and MB11_Dor2), Nude Metastasis (MB11_Nu1 and MB11_Nu2), and Overt Metastasis (MPA72_2A and MPA73_2A).


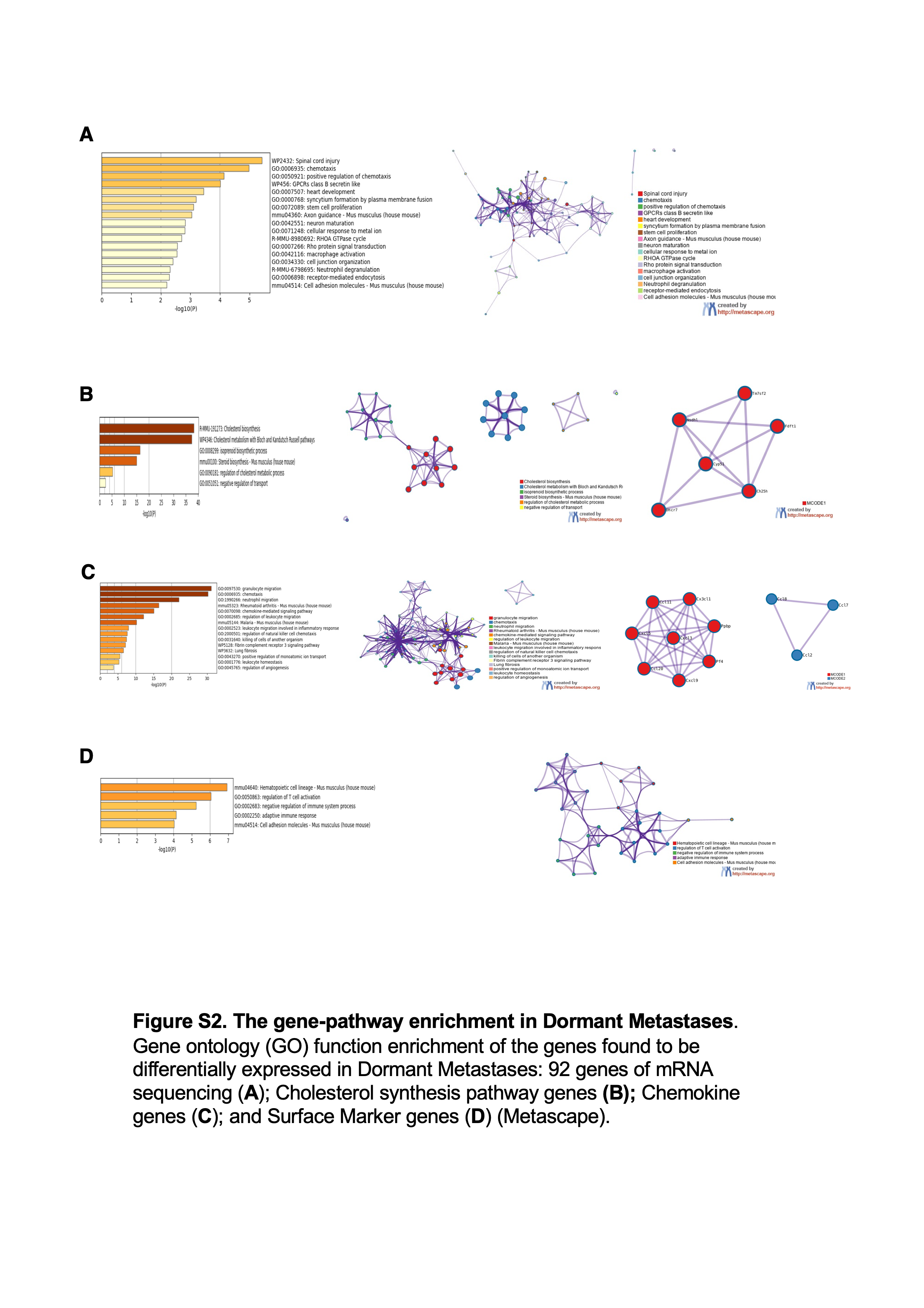


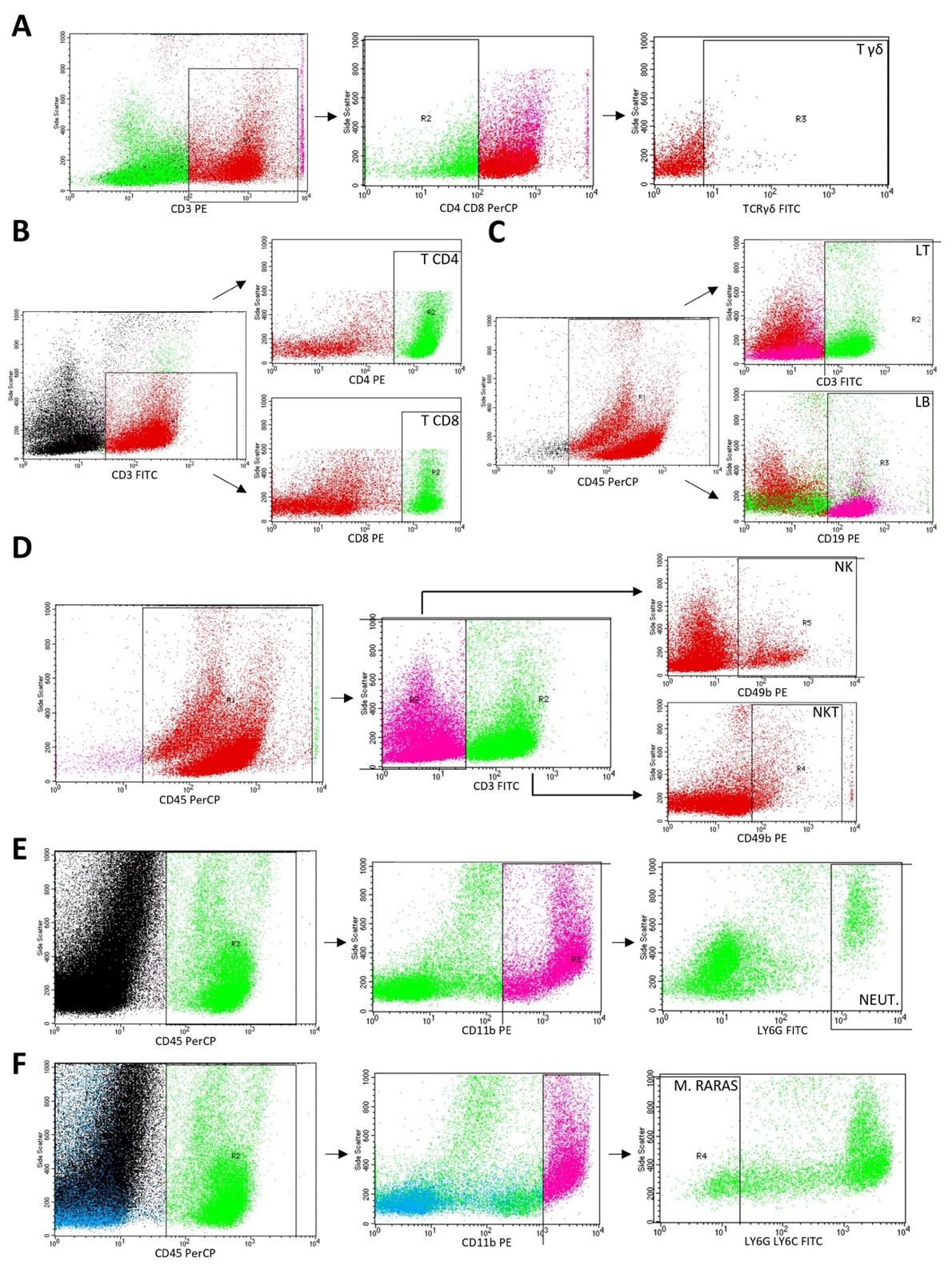


**Figure S3**. **Flow cytometry gating strategies:** a CD3+ x (CD4- CD8-) x TCRγδ+ gate was applied to select γδ T lymphocytes (**A**); CD3^+^ x CD4^+^ y CD3^+^ x CD8^+^ to classify CD4 and CD8 T lymphocytes (**B**); CD45^+^ x CD3^+^ y CD45^+^ x CD19^+^ to separate the T and B lymphocytes (**C**); CD45^+^ x CD3^-^ x CD49b^+^ y CD45^+^ x CD3^+^ x CD49b^+^ to differentiate between NK and NKT cells (**D**); CD45^+^ x CD11b^+^ x LY6G^+^ for selecting neutrophils (**E**), gate showing as a representation of eosinophils (CD11b^+^ x SIGLEC-F^+^), monocytes (CD11b^+^ x LY6C^+^), macrophages (CD11b^+^ x MHC-II^+^), interstitial macrophages (CD11b^+^ x F4-80^+^), alveolar macrophages (CD11c^+^ x SIGLEC-F^+^) and dendritic cells (CD11c^+^ x MHC-II^+^), in which the same gating strategy has been used but with its specific markers; and CD45^+^ x CD11b^+^ o CD11c^+^ x (LY6G^-^ LY6C^-^) to identify rare myeloid cells (**F**).

**Supplementary Tables**

**Supplementary Table S1.**

**Differentially expressed genes in Dormant metastases obtained by RNA-seq**

| Gene_id | | **Gene** | **Dormant-met** | **Nude-met** | **Over-met** |
| --- | --- | --- | --- | --- | --- |
|  | |  | log2*** | log2 | log2 |
| ENSMUSG00000028871 |  | **Rspo1** | 10,0 | 0,0 | 0,4 |
| ENSMUSG00000033788 |  | **Dysf** | 6,9 | 0,8 | 2,0 |
| ENSMUSG00000040345 |  | **Arhgap9** | 6,4 | 0,4 | 2,2 |
| ENSMUSG00000025888 |  | **Casp1** | 6,3 | 1,3 | 1,9 |
| ENSMUSG00000052572 |  | **Dlg2** | 6,3 | 2,4 | -0,1 |
| ENSMUSG00000074676 |  | **Foxs1** | 6,1 | 3,2 | 3,6 |
| ENSMUSG00000079262 |  | **Slco1a6** | 6,1 | 2,2 | 0,7 |
| ENSMUSG00000074457 |  | **S100a16** | 6,0 | 2,5 | 3,7 |
| ENSMUSG00000050370 |  | **Ch25h** | 5,6 | -5,7 | 0,0 |
| ENSMUSG00000031099 |  | **Smarca1** | 5,5 | 2,1 | -2,5 |
| ENSMUSG00000036446 |  | **Lum** | 5,1 | 2,8 | -5,3 |
| ENSMUSG00000058248 |  | **Kcnh1** | 4,6 | -3,1 | 1,2 |
| ENSMUSG00000006490 |  | **Prl8a9** | 4,4 | -3,5 | -4,5 |
| ENSMUSG00000025105 |  | **Bnc1** | 4,0 | -1,7 | -0,5 |
| ENSMUSG00000027070 |  | **Lrp2** | 4,0 | 1,3 | 2,0 |
| ENSMUSG00000022636 |  | **Alcam** | 3,9 | -3,4 | -4,2 |
| ENSMUSG00000070705 |  | **Eid2b** | 3,8 | -2,9 | 0,2 |
| ENSMUSG00000028370 |  | **Pappa** | 3,8 | -0,6 | -0,8 |
| ENSMUSG00000032322 |  | **Pstpip1** | 3,6 | 1,5 | 0,3 |
| ENSMUSG00000028391 |  | **Wdr31** | 3,6 | 0,4 | 1,9 |
| ENSMUSG00000017737 |  | **Mmp9** | 3,5 | 0,8 | -1,3 |
| ENSMUSG00000043557 |  | **Mdga1** | 3,4 | -1,6 | -4,5 |
| ENSMUSG00000098923 |  | **Tmem185b** | 3,3 | -4,5 | -4,4 |
| ENSMUSG00000002885 |  | **Cd97** | 3,1 | 0,4 | -2,5 |
| ENSMUSG00000039062 |  | **Anpep** | 2,9 | 1,5 | -0,4 |
| ENSMUSG00000092130 |  | **D030025P21Rik** | 2,9 | -0,7 | 0,3 |
| ENSMUSG00000024827 |  | **Gldc** | 2,9 | 1,0 | -1,4 |
| ENSMUSG00000099406 |  | **RP23-480D7.1** | 2,8 | -1,9 | -2,0 |
| ENSMUSG00000063975 |  | **Slco1a5** | 2,7 | -0,4 | -0,3 |
| ENSMUSG00000031647 |  | **Mfap3l** | 2,6 | 0,0 | -0,5 |
| ENSMUSG00000025473 |  | **Adam8** | 2,5 | -1,1 | 1,0 |
| ENSMUSG00000024899 |  | **Papss2** | 2,5 | -1,4 | -5,0 |
| ENSMUSG00000024778 |  | **Fas** | 2,3 | -5,7 | 0,2 |
| ENSMUSG00000002020 |  | **Ltbp2** | 2,2 | 0,1 | 0,6 |
| ENSMUSG00000046157 |  | **Tmem229b** | 2,2 | -0,8 | -0,7 |
| ENSMUSG00000038178 |  | **Slc43a2** | 2,2 | -0,4 | -0,5 |
| ENSMUSG00000055737 |  | **Ghr** | 2,2 | -0,7 | -2,2 |
| ENSMUSG00000027962 |  | **Vcam1** | 2,1 | 0,2 | 0,3 |
| ENSMUSG00000040466 |  | **Blvrb** | 2,0 | -0,5 | -0,1 |
| ENSMUSG00000017417 |  | **Plxdc1** | 2,0 | -1,3 | -5,6 |
| ENSMUSG00000031558 |  | **Slit2** | 2,0 | -0,9 | -2,8 |
| ENSMUSG00000035373 |  | **Ccl7** | 2,0 | -2,9 | -2,5 |
| ENSMUSG00000041439 |  | **Mfsd6** | 1,9 | -2,5 | -2,0 |
| ENSMUSG00000031827 |  | **Cotl1** | 1,9 | -0,3 | -2,2 |
| ENSMUSG00000059588 |  | **Calcrl** | 1,8 | -0,5 | -2,7 |
| ENSMUSG00000011148 |  | **Adssl1** | 1,7 | 0,1 | -3,6 |
| ENSMUSG00000020902 |  | **Ntn1** | 1,6 | 0,2 | 0,2 |
| ENSMUSG00000057933 |  | **Gsta2** | 1,6 | -1,5 | -0,9 |
| ENSMUSG00000035385 |  | **Ccl2** | 1,5 | -2,7 | -1,4 |
| ENSMUSG00000086320 |  | **Gm12840** | 1,4 | -1,1 | -0,9 |
| ENSMUSG00000021403 |  | **Serpinb9b** | 1,4 | -1,3 | -0,2 |
| ENSMUSG00000031762 |  | **Mt2** | 1,4 | -1,1 | -3,0 |
| ENSMUSG00000018604 |  | **Tbx3** | 1,4 | -1,2 | -2,9 |
| ENSMUSG00000032340 |  | **Neo1** | 1,3 | -0,7 | -1,0 |
| ENSMUSG00000057457 |  | **Phex** | 1,3 | -0,1 | -2,6 |
| ENSMUSG00000029108 |  | **Pcdh7** | 1,3 | -0,7 | 0,0 |
| ENSMUSG00000047878 |  | **A4galt** | 1,3 | -0,9 | -7,3 |
| ENSMUSG00000026094 |  | **Stk17b** | 1,2 | -0,9 | 0,0 |
| ENSMUSG00000022150 |  | **Dab2** | 1,2 | -0,3 | -3,7 |
| ENSMUSG00000021614 |  | **Vcan** | 1,2 | -1,6 | -6,5 |
| ENSMUSG00000072812 |  | **Ahnak2** | 1,2 | -0,5 | -0,6 |
| ENSMUSG00000032014 |  | **Oaf** | 1,1 | -0,9 | -1,0 |
| ENSMUSG00000017002 |  | **Slpi** | 1,1 | -2,2 | -3,0 |
| ENSMUSG00000037679 |  | **Inf2** | 1,1 | -0,8 | -0,2 |
| ENSMUSG00000055436 |  | **Srsf11** | -1,1 | 0,6 | 0,0 |
| ENSMUSG00000028180 |  | **Zranb2** | -1,2 | 0,5 | 0,1 |
| ENSMUSG00000039137 |  | **Whrn** | -1,2 | -2,7 | -3,8 |
| ENSMUSG00000024664 |  | **Fads3** | -1,4 | 0,1 | 0,2 |
| ENSMUSG00000049521 |  | **Cdc42ep1** | -1,9 | -0,3 | 0,6 |
| ENSMUSG00000024043 |  | **Arhgap28** | -1,9 | 1,0 | 1,2 |
| ENSMUSG00000028184 |  | **Adgrl2** | -2,0 | 0,5 | 1,0 |
| ENSMUSG00000028885 |  | **Smpdl3b** | -2,3 | 0,6 | 0,5 |
| ENSMUSG00000041642 |  | **Kif21b** | -2,4 | -0,3 | -0,2 |
| ENSMUSG00000024501 |  | **Dpysl3** | -2,5 | -0,6 | 1,3 |
| ENSMUSG00000027833 |  | **Shox2** | -2,6 | 0,0 | -0,3 |
| ENSMUSG00000054855 |  | **Rnd1** | -2,8 | -0,5 | 0,7 |
| ENSMUSG00000026640 |  | **Plxna2** | -3,1 | 0,2 | 1,1 |
| ENSMUSG00000003355 |  | **Fkbp11** | -3,9 | -0,4 | 1,2 |
| ENSMUSG00000041180 |  | **Hectd2** | -3,9 | 0,6 | 3,5 |
| ENSMUSG00000039405 |  | **Prss23** | -5,4 | 0,9 | 0,4 |
| ENSMUSG00000008035 |  | **Mid1ip1** | -5,7 | -0,3 | -1,4 |
| ENSMUSG00000022676 |  | **Snai2** | -6,2 | 0,0 | -0,1 |
| ENSMUSG00000079018 |  | **Ly6c1** | -7,0 | -1,1 | 0,9 |
| ENSMUSG00000075602 |  | **Ly6a** | -7,9 | -0,8 | 2,3 |
| ENSMUSG00000043415 |  | **Otud1** | -8,5 | -0,9 | 0,8 |
| ENSMUSG00000031523 |  | **Dlc1** | -8,8 | -0,9 | 2,0 |
| ENSMUSG00000031841 |  | **Cdh13** | -9,2 | -0,1 | 1,6 |
| ENSMUSG00000070867 |  | **Trabd2b** | -9,2 | -1,0 | 2,7 |
| ENSMUSG00000021913 |  | **Ogdhl** | -9,5 | -0,4 | 0,2 |
| ENSMUSG00000028173 |  | **Wls** | -9,9 | -0,8 | -1,3 |
| ENSMUSG00000047013 |  | **Fbxo41** | -10,0 | 1,2 | 1,6 |
| ENSMUSG00000028175 |  | **Depdc1a** | -10,0 | 1,0 | 0,0 |

*** p < 0.001 comparing Dormant-met group to the other two metastasis groups

**Supplementary Table S2.** **Gene identification from GO Database**

**Cholesterol biosynthesis**

**Chemokines**

**Surface Markers**
